# Surgical Management of L5-S1 Spondylodiscitis on Previously Documented Isthmic Spondylolisthesis: Case Report and Review of the Literature

**DOI:** 10.1155/2020/1408701

**Published:** 2020-02-15

**Authors:** Anthony Lubiato, Guillaume Baucher, Mikael Meyer, Stéphane Fuentes

**Affiliations:** Department of Adult Neurosurgery, La Timone University Hospital, APHM, Aix Marseille University, 264 Rue Saint Pierre, Marseille 13385, France

## Abstract

**Background:**

Although lumbar isthmic spondylolisthesis is frequent in the Caucasian population, its association with spondylodiscitis is extremely rare. *Case Description*. The authors reported the case of a 44-year-old patient affected by pyogenic spondylodiscitis on previously documented isthmic spondylolisthesis at the L5-S1 level. The patient was surgically treated by circumferential arthrodesis combining anterior lumbar interbody fusion (ALIF), followed by L4-S1 percutaneous osteosynthesis using the same anesthesia. Appropriate antibiotherapy to methicillin-susceptible *Staphylococcus aureus*, found on the intraoperative samplings, was then delivered for 3 months, allowing satisfactory evolution on the clinical, biological, and radiological levels. *Discussion*. This is the first case report of spondylodiscitis affecting an isthmic spondylolisthesis surgically treated by circumferential arthrodesis. In addition to providing large samplings for analysis, it confirms the observed evolution over the past 30 years in modern care history of spondylodiscitis, increasingly including surgical treatment with spinal instrumentation, thus avoiding the need of an external immobilization. Care must nonetheless be exercised in performing the ALIF because of the inflammatory tissue increasing the risk of vascular injury.

**Conclusion:**

Spondylodiscitis occurring on an L5-S1 isthmic spondylolisthesis can be safely managed by circumferential arthrodesis combining ALIF then percutaneous osteosynthesis in the same anesthesia, obviously followed by appropriate antibiotherapy.

## 1. Introduction

Spondylolisthesis and spondylodiscitis are nowadays common spinal pathologies in the neurosurgery department [1, 2]. Although spondylodiscitis is rare in the general population [3], its current increasing incidence is deemed to be multifactorial, due to the growth of ageing people and chronic diseases, improvement of diagnostic tools, general use of indwelling intravascular catheters, and immunosuppressive therapies [4]. Likewise, history of intravenous drug abuse [5] represents another major risk factor of pyogenic spondylodiscitis, the disruption of the cutaneous barrier causing direct blood contamination by pathogenic germs. The following hematogenous infection of vertebral endplates then spreads to the intervertebral disc space, finally resulting in the classical presentation of spondylodiscitis. On the other side, lumbar isthmic spondylolisthesis, caused by a defect in the *pars articularis*, is a common radiological entity and may be observed in up to 20% of spine X-rays, most of these cases being asymptomatic [6, 7]. While they are not usually associated, spondylolisthesis following spondylodiscitis is a rare and already described phenomenon [8]. Sometimes, the chronology may differ and infection occurs in a degenerative intervertebral disc. This case report is about a 44-year-old patient with L5-S1 pyogenic infection on a previous isthmic spondylolisthesis.

## 2. Case Presentation

**Figure 1:**
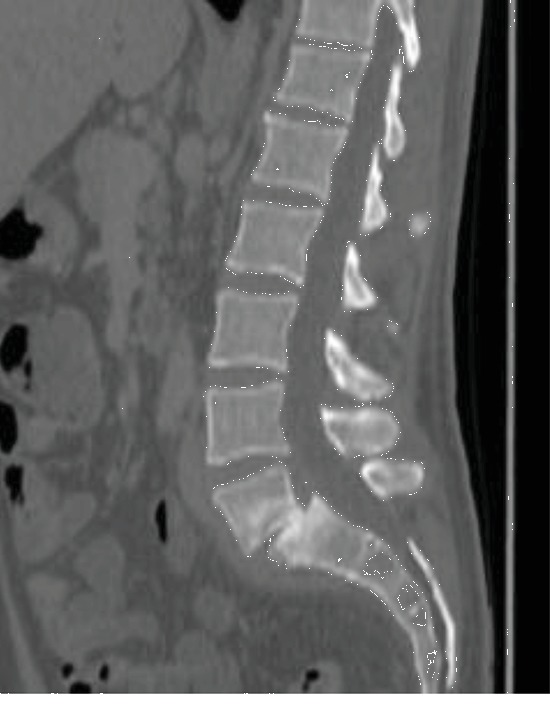
The lumbar spine in sagittal sections, highlighting an L5-S1 spondylolisthesis by isthmic lysis.

A 44-year-old patient, with a medical history of intravenous drug abuse and hepatitis C infection, was admitted for erysipelas of the left leg following cutaneous wound. Blood cultures grew methicillin-susceptible *Staphylococcus aureus*. Systematic thoraco-abdominopelvic CT scan was performed, demonstrating asymptomatic L5-S1 isthmic spondylolisthesis, without clear sign of spondylodiscitis (Figure 1).

**Figure 2:**
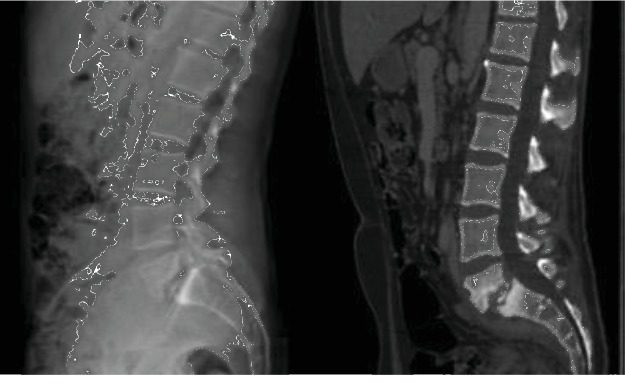
Preoperative (a) lumbar spine radiography in standing position and (b) lumbar CT scan, demonstrating the L5-S1 spondylodiscitis on previous spondylolisthesis, with major osteolysis of the vertebral endplates.

Intravenous antibiotic therapy with Orbenin was administrated for 2 weeks, with satisfactory biological and clinical evolution, allowing hospital discharge of the patient at the end of his treatment. Three weeks later, the patient is readmitted for low back pain and persistent fever. Neurological examination revealed intermittent bilateral L5 sciatica with drug-resistant pain. Lumbar MRI and CT scan demonstrated this time undoubted signs of L5-S1 spondylodiscitis on preexisting spondylolisthesis, with appearance of major mirror-image osteolysis of the vertebral endplates (Figure 2).

**Figure 3:**
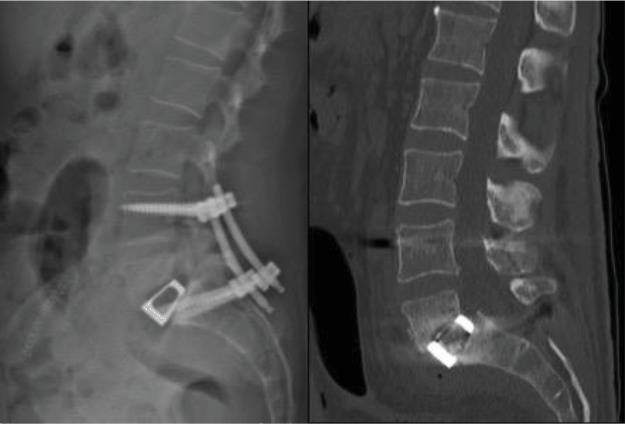
Postoperative (a) lumbar spine radiography in standing position and (b) lumbar CT scan, demonstrating the restoration of the lumbar lordosis and partial reduction of the L5-S1 spondylolisthesis.

The blood cultures were once again positive with methicillin-susceptible *Staphylococcus aureus*, and intravenous Orbenin was once more administrated. Because of the serious osteolysis, potential spinal instability threatening the neurological functions, and disabling pain, surgical treatment was decided, consisting of L5-S1 circumferential arthrodesis. Anterior lumbar interbody fusion (ALIF) of the L5-S1 level was first performed, using a Medtronic AVILA cage, with 16 degrees angulation and 10 mm height, to restore correct lumbar lordosis. Cancellous bone graft was placed in the cage to achieve the required fusion. Consecutive posterior percutaneous L4-S1 fixation was then completed, finalizing the stabilization, using the same anesthesia. The L5 level was willingly spared due to low radiographic visibility of the pedicles and significant osteolysis of the vertebra (Figure 3).

**Figure 4:**
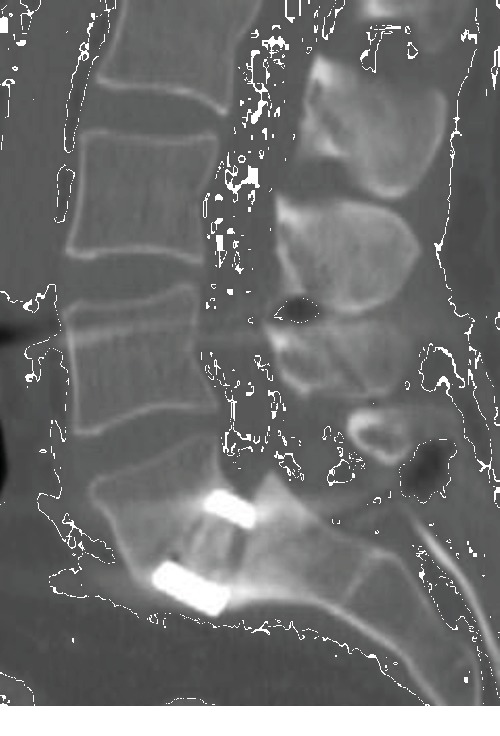
One-year CT scan control, highlighting the correct fusion of the L5-S1 level.

The intraoperative disc sampling appeared to be positive with methicillin-susceptible *Staphylococcus aureus*, in spite of ongoing intravenous antibiotic therapy. Modified treatment by Ofloxacin and Rifampicin, appropriate to the antibiogram, was delivered for 3 months after the surgery, with hospital discharge at J9. One year later, the infection was cured, and the patient did not complain about low back pain anymore and was able to return to work without difficulty, while the radiological assessment demonstrated satisfying fusion of the L5-S1 arthrodesis (Figure 4).

## 3. Discussion

### 3.1. A Rare Pathological Entity

Few reports are available about spondylodiscitis on previous isthmic spondylolisthesis, and none of them described surgical procedure. Tanaka et al. [9] mentioned the case of a 35-year-old orthopedic surgeon experiencing an L4-L5 spondylodiscitis, while this patient was already conservatively treated for a radiologically documented L4-L5 isthmic spondylolisthesis inducing chronic low back pain. Following medical treatment including culture-specific antibiotics and lumbar bracing, favorable outcome was achieved. Guglielmino et al. [10] related the only other adult case about a brucellar spondylodiscitis, with retroperitoneal muscle abscess surgically drained, but without spinal instrumentation and definitive radiological proof of a preexisting spondylolisthesis. The remaining subjects [11, 12] referred to pediatric patients for whom a nonoperative treatment was decided. Nevertheless, Nagashima et al. [12] featured the specificity of a spontaneous fusion of the grade III isthmic spondylolisthesis at the L5-S1 level. Considering that the annulus fibrosus is approximately supplied by vessels up to the age of 20 years [13, 14], the primary stage of infection is named discitis in this population, whereas the first step of infection in older patients is deemed to be vertebral osteomyelitis [15], with secondary spread to the intervertebral disc and adjacent vertebral endplate, justifying the term of spondylodiscitis. On the other hand, a histology study from Roberts et al. [16] pointed out the presence of blood vessels developing from the outer aspect of the annulus fibrosus in degenerative discs. Likewise, an immunohistochemical study from Ali et al. highlighted vascularization-related growth factor in the annulus fibrosus of degenerative discs, highly connected with the progression of angiogenesis [17]. These more recent findings are consistent with long-time observations [18], stating that pyogenic infections can primarily occur in the intervertebral disc, especially in degenerative levels, namely, spondylolisthesis in our example.

### 3.2. Surgical Management

Even though spondylodiscitis treatment first and foremost relies on appropriate antibiotic therapy and spinal immobilization, several cases require surgical interventions. Occurrence of a motor deficit by medullary or nerve root compression imposes urgent laminectomy for functional prognosis, with optional spine fixation by osteosynthesis [19]. The presence of an epidural abscess threatening the integrity of the dural sac also represents semiurgent surgical indication, taking account of the potential neurological compromise [20]. When bony destruction jeopardizes spine stability and clear local kyphosis is demonstrated, as for our case report, surgical spinal stabilization appears as a safe option [21], considering anterior, posterior, or combined approaches, adapted to the spinal level and degree of osteolysis. Anterior discectomy and fusion in septic condition with external spinal immobilization has been a long-term proposed therapeutic option [22–24] with satisfying fusion rates and absence of infectious recurrence. Nevertheless, one must specify that if vascular injuries are already common in ALIF in a noninfectious context [25, 26], debridement and bone grafting in discitis are often more challenging because of the inflammatory tissue, logically leading to an increased risk of vascular wounds [27]. Posterior instrumentation has also been proposed in case of vicious instability of the affected spinal region [28–30], avoiding in this way additional thoracolumbar bracing. Both sequential or simultaneous surgical strategies (using the same or two different anesthesia) have been compared without significant impact on the clinical outcomes [31, 32]. We advocated the simultaneous strategy with only one anesthesia, first performing the ALIF to optimize the opening of the disc space and reduction of the spondylolisthesis and to prevent the secondary migration of the bone graft [30]. In our specific case, the circumferential arthrodesis of L5-S1 was obviously the best surgical option because of the spinal instability caused by the major osteolysis of the vertebral endplates. Moreover, the use of a large cage, restoring disc height and thus bilaterally opening the intervertebral foramens, as well as the drainage of the intervertebral abscess, released the L5 roots. Association with effective antibiotherapy, decreasing of the local inflammation, allowed final bilateral L5 sciatica relief. Reduction of mobility induced by lumbar arthrodesis may possibly reduce axial pain [33], explaining the favorable evolution of our patient. Lastly, the increased risk of venous thromboembolism induced by immobilization and spinal surgery, especially concerning a patient with a history of intravenous drug abuse, justifies adapted prophylactic protocol [34]. Despite satisfying results one year after surgery, longer follow-up would be necessary to confirm proper bone healing and advanced fusion without infection recurrence, outlining the limits of a single retrospective case report.

### 3.3. Bacteriology

*Staphylococcus aureus* represents the causal infectious agent in 40 to 60% of cases of spondylodiscitis [3], the large majority of community-acquired strains being susceptible to methicillin [5]. However, it should be noted that the involvement of methicillin-resistant *Staphylococcus aureus* in spondylodiscitis is at the present time becoming more frequent [5]. In our case report, one specific interesting point is the positivity of the disc samples despite the beginning of an effective antibiotherapy before undergoing surgery. One can argue that discectomy and thus equivalent of surgical debridement have potentially facilitated the resolution of the infection in this particular case, by effective draining of the abundant pus. This intraoperative observation, comparable to an abscess with poor antibiotic diffusion, can explain the temporary resistance of infection to antibiotherapy at the time of surgery. Furthermore, if the blood cultures are negative at the initial assessment, the anterior discectomy with surgical samplings appears to be more effective than simple CT-guided biopsy to identify the pathogenic agent, getting more material for bacteriological and fungal analyses, especially following the beginning of probabilistic broad-spectrum intravenous antibiotics [35]. In our case, treatment failure at the initial stage of the infection (bacteremia following erysipelas) highlights the lack of sensitive and specific biomarkers of antibiotherapy effectiveness. In this context, current research on innovative blood sampling and analysis techniques for detection of biomarkers and microorganisms appears relevant for both initial diagnosis and monitoring of the response to treatment [36, 37]. Similarly, the negativity of CT scan emphasizes its lack of sensitivity at the early stages of spondylodiscitis, also demonstrated for MRI [38]. In this way, one can argue the usefulness of PET (positron emission tomography) CT scan in such presentation, because of its low likelihood ratio negative and performance at early stages of infection (even in the presence of spinal implant) [39].

## 4. Conclusion

Spondylodiscitis on previous isthmic spondylolisthesis is nowadays a rare, but an already known entity, most often conservatively treated. Here, we described the first case receiving a circumferential arthrodesis in this context of major osteolysis and consequent spinal instability. The favorable clinical and radiological evolution supports the idea that a justified instrumentation is safe in spinal infection.
